# Glocalizing the theory of learning: a pluriversal perspective on health professions education

**DOI:** 10.3389/fmed.2026.1929357

**Published:** 2026-09-04

**Authors:** Nabil Zary

**Affiliations:** Institute of Learning, Mohammed Bin Rashid University of Medicine and Health Sciences, Dubai Health, Dubai, United Arab Emirates

**Keywords:** artificial intelligence, curriculum, decoloniality, glocalization, health professions education, learning theory, pluriversality, professional identity formation

## Abstract

Glocalization has emerged as the preferred solution in health professions education (HPE) to the tension between globally recognized standards and local training practices. Its structure, however, is hub-and-spoke: theory is developed at a Western center, adaptation handled at the periphery. Curricula, accreditation, and assessment are adapted locally; learning theories are treated as universal and unchanging. This Perspective contends that the glocalization project remains incomplete and suggests how to finish it: by glocalizing the very theory of learning. Drawing on bibliometric evidence of concentrated knowledge production and thirty years of educational practice across four continents, I propose a pluriversal extension that integrates Islamic, Hindu, Confucian, Buddhist, and Ubuntu traditions as active sources of learning theory rather than as cultural contexts for imported ideas. To keep engagement rigorous rather than romantic, I outline a five-level comparison framework (vocabulary, phenomenon, mechanism, prescription, ontology), state its provenance and limits, and demonstrate its operation in worked examples and a complete supplementary application. The paper closes with theoretical and practical implications for curriculum, assessment, research capacity, and artificial intelligence, as well as an agenda for validating the framework.

## Introduction

Health professions education has spent twenty years establishing a global structure of shared competency frameworks, accreditation standards, and an expanding body of research (1). Alongside it has grown the recognition, named glocalization, that globally endorsed standards often align poorly with local training contexts (2–4). Accreditation has been adapted across East Asia (2), competency-based education has been localized into specific frameworks (4), and educators have been encouraged to move from globalization toward glocalization (3). This Research Topic asks how to prevent uncritical adoption of external models; taken seriously, the question reaches one level deeper: what constitutes importation when the imported element is a learning theory rather than a curriculum?

**Figure 1:**
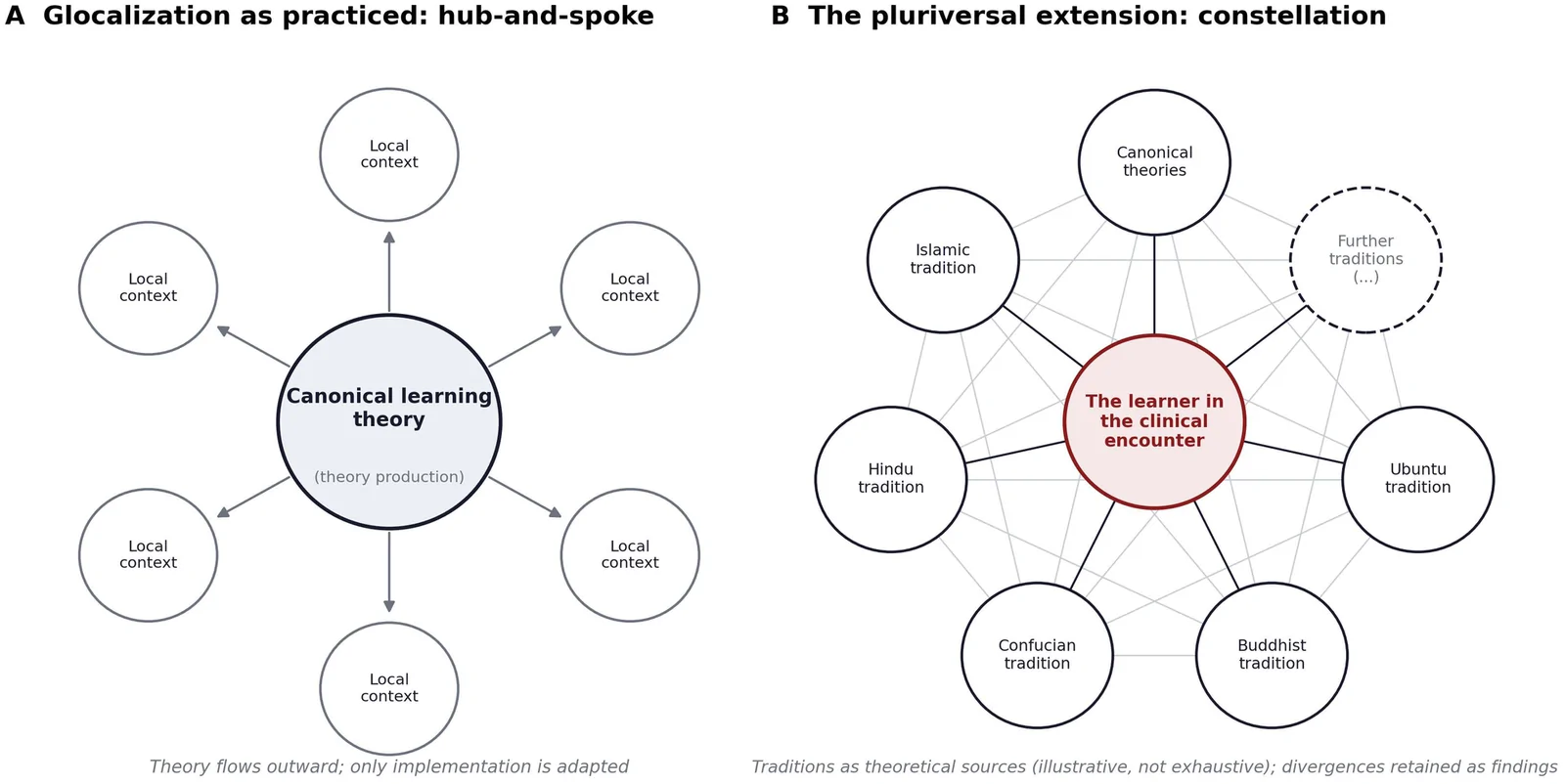
Two architectures for the relationship between learning theory and context in health professions education. **(A)** Glocalization as practiced: theory is produced by the canonical center and flows outward; local contexts adapt implementation. **(B)** The pluriversal extension: multiple intellectual traditions serve as theoretical sources around the learner in the clinical encounter; traditions are interconnected, and points of divergence among them are retained as theoretical findings rather than defaulting to the canon. The constellation is illustrative, not exhaustive.

To date, glocalization has employed a hub-and-spoke model (Figure 1A): the hub generates theory, the spokes handle adaptation. Localization occurs in implementation through curriculum maps, assessment blueprints, and accreditation crosswalks. The underlying learning theory remains unlocalized because it is never marked as local in the first place. The core theories supporting global standards (behaviorist, cognitivist, constructivist, humanist) travel as placeless science, yet they stem from specific intellectual traditions with particular views of what a learner is. The dominance is not merely impressionistic. United States and Canadian authors produced 95% of Academic Medicine articles between 1995 and 2000 (5), and five countries accounted for 74% of first and last authorships in the five leading medical education journals between 2012 and 2021, while 43% of the world's countries produced none (6). The theories share the same geography: the foundational statements of behaviorism, social cognitive theory, and experiential learning were authored within a single academic culture (7–9). Authorship concentration does not, by itself, prove theoretical dominance, but, when paired with critical analyses of the field's Northern theoretical tilt (10), it establishes the pattern this paper addresses.

These assumptions are significant. The traditional view of a learner in canonical literature depicts an individual as secular, autonomous, and psychologically motivated, making decisions on a private developmental path. Many learners worldwide, even within Western institutions, are shaped by traditions in which knowledge is understood as devotion, the teacher-student relationship is a moral covenant, and becoming a healer entails becoming a specific kind of person. When theory fails to recognize the learner, local curriculum adjustments cannot make the learner visible. Decolonial scholarship in health professions education (HPE) emphasizes this point: Bleakley and colleagues posed the post-colonial question nearly twenty years ago (11); Naidu argued that the core of medical education is a colonial construct (10, 12); Lokugamage and colleagues suggested engaging non-biomedical healing epistemologies on their own terms (13, 14); others critiqued the partnerships that maintain the imbalance (15, 16). This perspective introduces two key points: it shifts focus from curriculum and partnerships to the theory itself and advocates for a rigorous method to facilitate this shift.

The proposed framework is pluriversal. In decolonial scholarship, a pluriverse refers to “a world in which many worlds fit,” a concept from the Zapatista movement; it signifies a perspective where knowledge originates from multiple centers (17–20). In HPE, pluriversality involves engaging with major non-canonical traditions as sources of learning theory, each possessing its own mechanisms, institutions, and assessment practices (see Figure 1B). I write from a specific position: rooted in the Islamic intellectual tradition and the Western academic canon, as an outsider to the other traditions discussed, working within the publishing structures that this critique targets. What follows clarifies where I speak from personal formation and where from careful reading.

### What adaptation cannot fix

Canonical learning theories are not incorrect; they are partial, and that partiality is structural, recurring as a characteristic silence across every major family of the canon. One example in depth, then the pattern.

Self-determination theory explains motivation through three fundamental psychological needs: competence, autonomy, and relatedness (21). It is widely regarded as a highly effective framework in medical education (22), with extensive research on its cross-cultural applicability (23) and how religious values are internalized (24). The Islamic concept of talab al-ʿilm (knowledge-seeking as a religious duty) can be seen as either identified or integrated regulation, where an external value becomes internalized within the individual. Observe what the classification signifies; it is based on categorical differences, not degrees. Ontologically, the theory assumes a self that exists before its values and adopts them; in contrast, the tradition sees obligation as fundamental to what a learner is, implying that a value-free self cannot internalize values. Epistemologically, the theory views knowledge as an acquired resource, while the tradition regards knowledge-seeking as an act of worship, with learning defined through theological terms. Axiologically, the theory bases motivation on individual well-being; the tradition, however, grounds it in a moral order that transcends the self. “Identified” and “integrated” regulation do not simplify talab al-ʿilm; instead, they translate it into a framework whose core ontology it rejects. The Confucian pursuit of becoming an exemplary person, the Hindu linking of vocation with dharma, and the Ubuntu view of the person through community all resist direct translation. Self-determination theory does not poorly characterize such learners; it simply does not capture what is most profound within them.

The same pattern of silence persists throughout the canon: Behaviorism accounts for skill development but remains silent on the significance of what is shaped; social cognitive theory describes how learners emulate observed behaviors but does not explain what makes a model worth copying; and experiential learning outlines the cycle of experience and reflection but does not specify the ultimate goals of this cycle. Situated learning describes the social structure involved in clinical formation (25), with Wenger emphasizing the importance of identity (26). However, he remains silent on what constitutes the normative content of identity. Reflective practice (27) and transformative learning (28) clarify how perspectives evolve, while Mezirow's approach stems from Freire's emancipatory pedagogy (29), highlighting that the canon contains internal critics aligned with this perspective. Cognitive load theory enhances instruction without identifying the origins of the cultural content within schemas (30). Assessment science advanced through Miller's pyramid over thirty years (31), added the “is” level of professional identity a decade ago (32), while literature on the hidden curriculum admits that formation mostly happens outside the assessed curriculum (33). Validity theory points out what rubrics miss: construct underrepresentation (34), but simply naming underrepresentation does not explain what was underrepresented. Each omitted aspect follows the same pattern: a learning mechanism is described while the framework linking learning to formation is left unexamined. This shared omission represents the structural limitation that the pluriversal extension aims to address.

The missing material isn't unfamiliar to the West, which emphasizes monastic pedagogy, Bildung, and, closely related to medicine, Pellegrino and Thomasma's virtue ethics of clinical practice (35). Their exclusion from mainstream learning theory indicates a disciplinary and civilizational bias: twentieth-century psychology's focus on secular methods obscured formation-centered perspectives, even in Western contexts, highlighting the need to diversify the theoretical basis.

These costs (32–34) are well-documented and tangible. An altered memory from the early 2000s, modified to protect identity, depicts what these costs look like in a real room. In a European simulation center, a final-year student executed a perfect standardized patient exam, receiving top marks. The standardized patient, an experienced actress, unexpectedly wept afterward. This incident was not addressed in the debrief, as the rubric lacked a place to record such an emotional response. What the tears likely indicated was that the encounter was a moral and relational event for the patient, yet the evaluation scored it solely on technique. This anecdote illustrates a point, but the true evidence lies in the literature cited earlier. Over the past twenty years, I have concluded that this is not a failure of assessment but a failure of theory. The frameworks present could identify the gap but couldn't specify what should fill it. Adaptation alone cannot fix this because the problem is rooted in the underlying theoretical framework, not the instrument itself.

### Traditions as theory

The pluriversal claim holds that traditions regarded by the field as cultural contexts actually contain comprehensive learning theories. These include systematic explanations of how expertise is developed, institutional structures shaped over centuries, and assessment practices. Five traditions are analyzed based on four criteria: documented, long-standing institutional pedagogy; extensive literature supporting their claims; ongoing relevance in regions where many health professionals are trained; and prior mention in health professions education (HPE) scholarship. This selection is meant to illustrate rather than exhaustively represent all traditions; other systems, such as Daoist, Māori, Indigenous American, Latin American, and African knowledge, also meet these criteria and deserve engagement, guided by their own scholars (36). Each tradition is internally diverse and contested; what follows provides entry points rather than definitive summaries.

The Islamic tradition that shaped me supported an educational civilization with well-documented institutions (37, 38) and pioneering theorists who wrote comprehensive educational theories (39). Notable figures include al-Ghazali, who discussed the transformation of the knower (40); Ibn Khaldun, who examined the social conditions necessary for acquiring skills (41); and al-Ruhawi, who viewed the physician's intellectual, moral, and practical development as interconnected (42). Jurists regarded medicine as fard kifaya, a communal obligation: if no one in the community learned it, everyone was responsible, providing a theological basis for cultivating healers (39). Its primary credential, the ijazah, was primarily relational rather than psychometric, an endorsement from a designated teacher, based on long-term engagement (mulazama, suhba), that assured trust in the student's knowledge. The records also reveal its pitfalls: issuance of ijazahs to children and via correspondence, along with modern concerns over devaluation (38). These points are significant because the tradition has independently redefined relational credentialing as entrustment (43). Moreover, the ijazah serves as a millennium-long natural experiment in entrustment-based assessment, including issues of inflation and favoritism. Lessons that could inform modern fields piloting entrustable professional activities.

I approach the other four traditions as a careful outsider. The Hindu triad of śravaṇa, manana, and nididhyāsana (which means disciplined hearing, critical reflection, and meditative integration) originates from the Bṛhadāraṇyaka Upaniṣad (44) and is later systematized in Vedanta; its purpose is soteriological rather than professional, and this difference is itself insightful. Its residential teaching method made the teacher's cultivated life the core of the curriculum (45), and its medical writings contained explicit pedagogies for physicians (46). The Confucian tradition rejects the idea that competence and character can be separated in the canon: the ideal person (junzi) is formed by combining skill and virtue, cultivated through ritual and long-term observation of role models (47–49). The Buddhist tradition considers compassion (karuṇā) and attention as skills that can be developed through specific, step-by-step teachings (50, 51). Its concept of sati is more complex and unusual than the English term “mindfulness” (52), and Western clinical training is increasingly empirically investigating the idea that these qualities can be cultivated (53). The Ubuntu tradition deserves more in-depth treatment than the brief overview HPE provides. Ramose proposes an ontology where personhood is created through relations rather than residing within individuals (54). Metz advances this idea into a systematic relational moral framework (55). This has clear implications for learning theory: knowledge is produced and validated by the community; education is a collective process rather than a solitary journey; and evaluating a healer involves the community's recognition of relational behavior. The extent of this concept remains actively debated within African philosophy (56), and such debate is part of ongoing engagement. The varying depth of discussion across these paragraphs reflects my own development, as outlined in the Introduction. Outsider perspectives are tentative starting points, awaiting genuine insider perspectives.

A strict adherence to these traditions also involves rigorously enforcing their exclusions. For example, madrasa culture prevented women from becoming professors, despite women transmitting hadith with their own ijazahs (38). Vedic studies were limited by caste and gender, affecting South Asian medical education today. The Confucian exams were inaccessible to women, and the Pali Vinaya places nuns' orders under monks' authority. While a tradition's history can be compelling and influential, it often also entails exclusion. Critically engaging with these traditions as theoretical frameworks involves examining them carefully, just as one critiques the canon.

Today, these traditions are incorporated into HPE primarily as cultural competence or cultural safety, focusing on facts about patients and learners that need accommodation (57, 58). The pluriversal perspective shifts their role from being merely context to becoming a theoretical framework. This change is significant because theory influences curriculum design, assessment methods, and researcher training. Essentially, what is placed in the theoretical position dictates the field's perspective and understanding.

### Rigor, not romance

The primary critique is that this program encourages romanticism: it draws selective parallels, reduces traditions to mere precursors of Western thought, and fosters a reverence that isn't necessary for serious traditions. This concern is valid, which is why the program requires discipline as rigorous as any psychometric analysis.

I suggest defining any claimed convergence between traditions across five levels: vocabulary (whether terms translate), phenomenon (the observable process), mechanism (the explanation), prescription (the recommended practices), and ontology (their account of reality). The framework draws from three sources: the practice in comparative philosophy of identifying levels where traditions can genuinely agree; decolonial translation theory's emphasis that comparison must avoid imposing one side's categories as the standard (18); and decades of cross-traditional teaching, which showed that simplified comparisons often failed. This is a conceptual tool, not yet validated: its reliability will be determined through application studies, independent scholar use, and examining cases where levels conflict. Two examples demonstrate its discriminative ability; Supplementary Table S1 shows the full level-by-level application for replication by others.

Initially, there's a high-level convergence and a low-level divergence. Ibn Khaldun's view on skill acquisition introduces the concept of malaka: a sedimented habitus formed through practice, most reliably transmitted personally, as discussed in the Muqaddimah's sections on crafts and instruction (41). This aligns with situated learning (25) in terms of phenomenon and mechanism: both emphasize that expertise develops through ongoing participation in a practicing community. However, they differ fundamentally in ontology: Ibn Khaldun adopts a realist stance on collective life, unlike practice theory. Acknowledging this divergence isn't a concession; it's an essential insight.

Second, a false friend that fails at the first level is the Pali term 'sati,’ commonly translated as ‘mindfulness.’ It is inherently linked to memory and recollection, not just the present-moment awareness emphasized in secular programs (52). Therefore, a curriculum teaching “mindfulness” as Buddhist engagement is effectively using a translation artifact. The discipline recognizes both errors: the exaggerated claims and the false friend.

The discipline also addresses the projection objection. The concern that a reader recognizes their own tradition regardless of where they look. The solution is sequence: each tradition should be read first on its own terms, through its literature and, whenever possible, with scholars embedded within it (36), before making comparisons. de Sousa Santos refers to this comprehensive approach as intercultural translation (18). This method applies here: a single-authored description of five traditions is provisional; the entries in Table 1 are hypotheses awaiting insights from insiders, and the five levels themselves can be revised within this framework of translation.

**Table 1:** Five intellectual traditions serve as sources of learning theory for health professions education. Each row condenses an internally contested, historically layered tradition; entries are entry points for engagement, not summaries, and are offered as hypotheses awaiting theorization by scholars formed within each tradition. Entry points refer to the reference list.

Tradition	Core pedagogical concepts	What it theorizes that the canonical HPE literature does not	Entry points
Islamic	*talab al-ʿilm* (knowledge-seeking as obligation); *mulazama/suhba* (prolonged attachment to a teacher); *ijazah* (relational credential, ideal-typical form); *malaka* (habitus)	Motivation grounded in ultimate concerns; relational entrustment with documented failure modes; formation of intellect, character, and practice as one process	(37–43)
Hindu	*śravaṇa–manana–nididhyāsana* (hearing–reflection–integration; Vedantic systematization); *gurukula* residence; *dharma*-aligned vocation	A reception–reflection–integration cycle whose soteriological aim marks an instructive ontological divergence; the teacher's formed life as curriculum	(44–46)
Confucian	*junzi* (exemplary person); *ren* (integrated humaneness); *li* (ritual propriety)	Non-separability of competence and character; assessment of formation through extended observation of conduct	(47–49)
Buddhist	*karuṇā* (compassion); *sati* (attention-with-recollection, imperfectly rendered “mindfulness”); *sangha* (monastic training community)	Compassion and attention as trainable capacities with explicit graduated pedagogies	(50–53)
Ubuntu	Personhood constituted through relation; communal generation and validation of knowledge (scope internally debated)	Learning and healing as properties of communities, not only of individuals	(54–56)

Three limitations of this approach should be acknowledged. First is epistemic relativism: when every tradition serves as a source of theory, what basis is there for evaluating claims? Pluralism about sources clarifies disagreements rather than eliminating them. The five levels help specify disagreements without erasing them, and claims remain subject to each tradition's standards of rigor and overall empirical constraints in clinical education. Second, ontological incommensurability. Some perspectives on reality cannot be reconciled. The fifth level acknowledges incommensurability as an outcome rather than a problem to be resolved, and clinical practice already functions amid unresolved ontological debates. Third, ethical conflict. Moral commitments within traditions, including their exclusions, may conflict with each other and with professional responsibilities. Patient safety and the equal moral value of learners are essential standards; any irremediable conflict with these standards represents a level-four or level-five issue that limits transferability. The framework helps navigate these tensions but does not eliminate them.

## Discussion

The theoretical implications are prioritized first. The pluriversal extension does not replace canonical theories but repositions them, integrating them into a broader constellation. Initially invisible when isolated, their boundary conditions become researchable, as demonstrated by the self-determination analysis. This approach provides decolonial HPE literature (12, 13) with a practical method for conducting theory pluriversally, moving beyond mere calls for it. Additionally, it shifts the concept of glocalization from a one-way adaptation of existing theories to a bidirectional translation between traditions. This redefinition impacts what constitutes theoretical work and who is authorized to perform it. Four practical steps follow, each offering institutions actionable steps within this year.

### Curriculum

A pluriversal approach does more than just include a diversity module; it explicitly acknowledges its theoretical sources in a pluralistic manner. This is done within the same core courses and using the same critical framework as the traditional canon. Theories are chosen based on their relevance. For instance, a communication and compassion course could be structured around Buddhist graduated cultivation pedagogy (50, 51), taught alongside, not below, the mindful practice literature (53), with a clear distinction made between sati and mindfulness (52). The initial step is to review foundational HPE courses and identify the traditions they represent.

### Assessment

If professional identity formation is the core of Miller's updated pyramid (32), then the longstanding traditions of relational assessment serve as valuable resources. These traditions include documented failure modes that foreshadow the halo and leniency issues commonly seen in workplace-based assessments. A practical, testable protocol would involve structured longitudinal supervisor attestations on entrustment patterns (43), similar to the ijazah, using patient narratives as evidence rather than satisfaction scores, along with triangulated self-narratives. Two essential safeguards must be maintained: co-designing with scholars rooted in the original tradition and being open to the possibility that a relational covenant cannot be fully captured by psychometric measures. Without these safeguards, efforts risk becoming mere extraction. Using the tradition to generate a tool while ignoring the lived experience that gives it meaning (59). The initial step involves one clerkship over one year, with rigorous validity research (34).

Research capacity and its political economy are crucial. The program depends on whether scholars trained in these traditions can develop theories within the field's journals, rather than merely applying external frameworks to local populations (10, 14). Authorship data illustrate this baseline: five countries account for three-quarters of the field's leading authorship (6). Without diverse theoretical foundations, the infrastructure remains monocultural: accreditation requirements, licensing exams that enforce epistemic standards, article-processing fees that exclude necessary scholars, and English as the gatekeeper language. All sustain the coloniality of power (17). Initial steps include fostering bidirectional mentorship between early-career researchers and senior scholars rooted in these traditions, as well as editorial policies that support rigorous, methodologically pluralistic approaches.

### Artificial intelligence

Large language models perform credibly on the assessments that define competence (60) while reproducing the biases and gaps of their training data (61). The bias is both cultural and clinical: leading models tend to reflect the values of English-speaking and Protestant-European countries, and prompting only partially mitigates this skew (62). Since training corpora mainly consist of the field's published literature, a theoretical monoculture becomes an ingrained part of the tools that future generations will use. AI acts as a mirror, reflecting both the contents and omissions of the field's literature. The framework plays a clear role as HPE integrates AI (63): traditions serve as criteria for evaluating training data representation, the five levels function as a rubric for assessing whether an AI tutor's explanation of learning aligns with a tradition's perspective or is presented as universal, and corpus diversification acts as an intervention to promote epistemic justice. Begin by auditing which traditions are included in any AI educational tool the institution uses.

Glocalization was the correct instinct. Global frameworks should respond to local realities. However, this instinct stops short; completing it involves engaging traditions as a theoretical discipline, establishing a system for comparing them, and creating an infrastructure that allows local scholars to conduct theorizing. The research agenda includes validating the five-level framework via application studies and independent scholarship across different traditions; documenting each tradition's perspective on clinical formation led by insiders; developing and testing assessment tools based on relational-credential principles; conducting comparative studies of formation outcomes in pluriversal versus monocultural curricula; and auditing AI training datasets to ensure they align with diverse learning traditions. Completion is an accurate term. Fields expand on their existing commitments, but the extension reaches farther from the center than where it started. The real question is not whether canonical frameworks shift, but what the field resembles when it no longer has a single focal point.

## Data Availability

The original contributions presented in the study are included in the article/Supplementary Material, further inquiries can be directed to the corresponding author.
