# Supplementary material for: Glocalizing the theory of learning: a pluriversal perspective on health professions education
Source: Front Med (Lausanne). 2026 Sep 4;13:1929357. doi: 10.3389/fmed.2026.1929357 (PMC13585607; doi:10.3389/fmed.2026.1929357)
Supplement: Supplementary file 1 [file Table1.docx]

**Supplementary Material**

**Glocalizing the theory of learning: a pluriversal perspective on health professions education**

Nabil Zary, Institute of Learning, Mohammed Bin Rashid University of Medicine and Health Sciences, Dubai Health, Dubai, United Arab Emirates

# Supplementary Table S1

# Complete the application of the five-level comparison framework to the two worked examples in the main text. Each level is applied in sequence; the verdict at each level is independent, and the overall verdict reports the highest level at which convergence holds and the level at which it breaks. The framework treats divergence as a documented finding, not a failure of comparison. Reference numbers refer to the main text's reference list.

| **Level** | **Guiding question** | **Example 1: malaka (Ibn Khaldun) ↔ situated learning theory (Lave and Wenger)** | **Example 2: sati (Pali Canon) ↔ mindfulness (secular clinical programs)** |
| --- | --- | --- | --- |
| 1. Vocabulary | Do the core terms translate without remainder? | Partial but workable. Malaka translates as habitus or sedimented aptitude; no single Khaldunian term maps to "legitimate peripheral participation," but the semantic field of apprenticeship terms (talqin, direct transmission; suhba/mulazama, prolonged attachment) covers the relational core. Verdict: PASSES with documented remainder. | Fails. Sati derives from smrti (memory, recollection) and canonically denotes attention informed by remembering the teaching; "mindfulness" in secular programs denotes bare, non-judgmental present-moment attention (46). The shared English word conceals a semantic substitution. Verdict: FAILS. Comparison below this level inherits the artifact. |
| 2. Phenomenon | Are both traditions describing the same observable process? | Yes. Both describe the acquisition of durable practical expertise by novices through prolonged engagement in a community of skilled practitioners (20, 35). Verdict: PASSES. | Only partially. Both describe trained attention, but the canonical phenomenon includes ethical recollection and the secular phenomenon is defined to exclude it. Verdict: PARTIAL. |
| 3. Mechanism | Do they explain the process the same way? | Substantially. Ibn Khaldun: repeated practice under direct personal transmission sediments aptitude, with the surrounding social order (sedentary institutions, continuous transmission) as enabling condition. Lave and Wenger: legitimate peripheral participation with increasing centrality in a community of practice. Both mechanisms are participatory, relational, and gradual. Verdict: PASSES. | No. Canonical cultivation of sati proceeds within the graduated path: ethical restraint, guarding of the senses, and recollection of the teaching jointly produce right mindfulness (44, 45). Secular programs specify repeated present-moment attention exercises over a fixed course. The causal stories differ in both ingredients and ordering. Verdict: DIVERGES. |
| 4. Prescription | Do they recommend the same practices? | Largely. Both prescribe apprenticeship: prolonged attachment to accomplished practitioners, sequenced exposure to authentic tasks, and institutional conditions that sustain transmission. Differences are of emphasis (Ibn Khaldun stresses the master relation; situated learning stresses the community). Verdict: LARGELY PASSES. | No. The tradition prescribes graduated cultivation with ethical prerequisites within a formed community (45); secular programs prescribe a self-contained short course without ethical prerequisites. Verdict: DIVERGES. |
| 5. Ontology | Do their underlying accounts of reality align? | No. Ibn Khaldun's science of civilization is realist about collective entities: social cohesion and the crafts are features of a real collective order. Practice theory declines such commitments. Verdict: DIVERGES and the divergence is itself a theoretical resource: two accounts of what a "community" is, each researchable. | No. Canonical sati operates within a soteriological framework (liberation from suffering, non-self); secular mindfulness operates within therapeutic naturalism. Verdict: DIVERGES. |
| Overall verdict | — | Convergence holds at phenomenon, mechanism, and prescription; breaks at ontology. Implication: the traditions can co-inform clinical apprenticeship design while retaining distinct, documented accounts of what community is. | Convergence fails at vocabulary; apparent convergence below is an artifact of translation. Implication: curricula must either teach secular mindfulness as its own practice or engage Buddhist pedagogy on its own terms; the shared word licenses neither. |

# Note on replication. To apply the framework to a new concept pair: (1) establish each concept within its own tradition's literature before comparison, wherever possible with scholars formed in that tradition; (2) apply the five levels in order, recording a verdict and the textual evidence at each level; (3) report divergences with the same care as convergences. A documented divergence is a finding; (4) when levels give conflicting verdicts, report the conflict rather than forcing a summary judgment. Such boundary cases are the primary route by which the framework itself will be tested and revised.
